# The treatment of scaphoid nonunion using the Ilizarov fixator without bone graft, a study of 18 cases

**DOI:** 10.1186/1749-799X-6-57

**Published:** 2011-11-08

**Authors:** Marko Bumbaširević, Slavko Tomić, Aleksandar Lešić, Vesna Bumbaširević, Zoran Rakočević, Henry D Atkinson

**Affiliations:** 1 Institute for Orthopaedic Surgery and Traumatology, Clinical Center of Serbia, Belgrade, Serbia; 2 Institute for Orthopaedic Surgery "Banjica", Mihajla Avramovica 28, Belgrade, Serbia; 3 Department of Trauma and Orthopaedics, North Middlesex University Hospital and London Sports Orthopaedics, Sterling way N18 1QX, UK

**Keywords:** Scaphoid nonunion, Ilizarov circular frame, without bone graft

## Abstract

**Objectives:**

Evaluating the safety and efficacy of the Ilizarov fine-wire compression/distraction technique in the treatment of scaphoid nonunion (SNU), without the use of bone graft.

**Design:**

A retrospective review of 18 consecutive patients in one centre.

**Patients and Methods:**

18 patients; 17 males; 1 female, with a mean SNU duration of 13.9 months. Patients with carpal instability, humpback deformity, carpal collapse, avascular necrosis or marked degenerative change, were excluded. Following frame application the treatment consisted of three stages: the frame was distracted 1 mm per day until radiographs showed a 2-3 mm opening at the SNU site (mean 10 days); the SNU site was then compressed for 5 days, at a rate of 1 mm per day, with the wrist in 15 degrees of flexion and 15 degrees of radial deviation; the third stage involved immobilization with the Ilizarov fixator for 6 weeks. The technique is detailed herein.

**Results:**

Radiographic (CT) and clinical bony union was achieved in all 18 patients after a mean of 89 days (70-130 days). Mean modified Mayo wrist scores improved from 21 to 86 at a mean follow-up of 37 months (24-72 months), with good/excellent results in 14 patients. All patients returned to their pre-injury occupations and levels of activity at a mean of 117 days. Three patients suffered superficial K-wire infections, which resolved with oral antibiotics.

**Conclusions:**

In these selected patients this technique safely achieved bony union without the need to open the SNU site and without the use of bone graft.

## Introduction

First described by Causin and Destor in 1895, injuries to the scaphoid account for 70% of all carpal fractures [1], and with appropriate initial treatment the majority unite without complication [2,3]. However up to 45% of these fractures [4,5], often those occurring in young active patients [6], progress to a nonunion. The most common causes of scaphoid nonunion (SNU) relate to inadequate fracture immobilization (in terms of duration and type of immobilization), patient non-compliance with treatment, misdiagnosis, fracture displacement and associated carpal instability [3,7,8]. When SNU occurs it may initially show few symptoms, however it eventually leads to degenerative disease with arthritic changes in the scaphoradial, scaphocapitate and capitolunate joints, and around the radial styloid. Wrist joint function subsequently becomes limited, and often has a significant impact on the activities of daily living and the ability to work [6]. It has thus been advised to treat SNU early (within 12 months of injury) [3,9,10].

There is still no accepted "gold standard" for the treatment of SNU, and failures occur in up to 25% of cases [3,10]; influencing factors include: the time elapsed since injury, the type of operative treatment, the anatomical location of the SNU (i.e. the proximal pole), the development of scaphoid avascular necrosis (AVN), having had a previous styloidectomy (1), and the presence of a scaphoid humpback deformity [11]. SNU treatment options are:(i) fracture fixation alone, without bone grafting [12]; (ii) the use of non-vascularized bone grafting without internal fixation [13,14]; (iii) non-vascularized bone grafting with internal fixation [3,7,15-17]; (iv) the use of vascularized bone grafts, with or without internal fixation [18-20], with a recent systematic review reporting union rates of 80% using bone graft without fixation, 85% using bone graft with fixation, and 91%-100% using vascularized bone grafts [4,13,19].

Ilizarov fine-wire external fixation techniques have been used successfully in recalcitrant chronic long-bone nonunions. Bony healing is achieved though the application of compression and distraction at the fracture sites which is thought to improve local micro-circulation [20-24].

The aim of this study is to examine the efficacy and safety of SNU treatment using the Ilizarov compression/distraction technique without opening the SNU site and without the use of bone graft.

## Patients and methods

Eighteen patients with SNU treated between 2002 and 2006 were included in this retrospective review. Ethical approval was given by the Ethics Committee of Belgrade University, Serbia, and all the patients gave their informed consent for this study.

SNU was established when there was no progression in bony healing between 3 successive monthly radiographs (allowing a minimum of 6 months to elapse following injury) [3]; acknowledging that other imaging modalities such as MRI may be a more sensitive way of both diagnosing the fractures and gauging proximal pole vascularity [18]. SNU patients with (Dorsal Intercalated Segment Instability (DISI)) carpal instability, humpback deformities, carpal collapse due to AVN, or with marked degenerative changes were excluded, as these associated pathologies can negatively impact on surgical outcomes, and we felt that the selected patients would be the most ideal for pilot-testing this new technique. Scapholunate and other ligament assessments were made under anaesthesia checking for carpal instability.

The series included seventeen male patients and one female with a mean age of 23.5 years (15-34 years) and all with their dominant hands affected (17 right and 1 left). Six patients were professional sportsmen, three were office workers who regularly played sports, four were manual laborers, four were students, and one was unemployed. Six patients were smokers; though no patient smoked during the duration of treatment.

The initial scaphoid fracture resulted from a sporting accident in nine patients, from falls in five, and one patient sustained his injury by punching a wall. Fourteen patients had been initially treated in below-elbow "scaphoid" plaster-cast immobilization: five patients for 8 weeks, four for 10 weeks, one for 11 weeks, one for 14 weeks, one for 15 weeks, one for 16 weeks and one for 18 weeks); four patients had received no initial treatment, due to late presentations.

**Figure 1:**
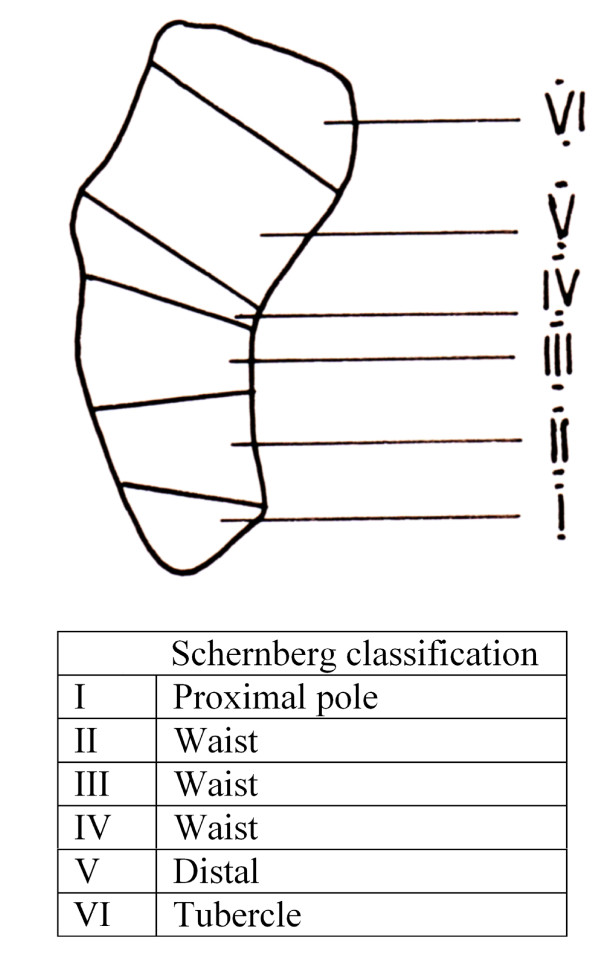
**Schernberg's scaphoid classification (32) (I-proximal pole, II, III, IV-waist, V-distal and VI-tubercle)**.

The mean duration of SNU at Ilizarov frame application (index procedure) was 13.9 months (range 7-36 months). The location of the SNU was in the waist of the scaphoid (zone II, III, IV) in 14 patients, the proximal pole (zone I) in three patients, and in the distal scaphoid (zone V) in one patient, according to Schernberg's classification [25] (Figure 1**)**. Mild degenerative changes were noted in two cases. Scapholunate and capitolunate angles, and the carpal height index were assessed both pre and postoperatively [26].

Patient demographics, occupations, sporting activities, mechanisms of injury, and duration and types of SNU are shown in Table 1.

**Table 1:** Scaphoid non-union (SNU) pre-operative patient data.

Case	Sex	Age	Occupation/Sports activity	Mechanism of injury	Side	Duration of SNU (months)	SNU type*
1	M	15	Basketball	Sport	Left	21	IV

2	M	21	Waterpolo	Sport	Right	24	III

3	M	20	Waterpolo	Sport	Right	36	III

4	M	27	Manual Laborer	Punching a wall	Right	15	III

5	F	27	Basketball	Fall	Right	7	IV

6	M	26	Student	Sport	Right	8	IV

7	M	27	Student	Sport	Right	36	IV

8	M	22	Office/Volleyball	Sport	Right	9	III

9	M	25	Manual Laborer	Fall from a height	Right	6	III

10	M	34	Manual Laborer	Fall	Right	12	III

11	M	27	Unemployed	Fall	Right	6	V

12	M	18	Goalkeeper	Sport	Right	12	I

13	M	21	Student	Sport	Right	24	III

14	M	22	Student	Fall	Right	7	IV

15	M	24	Office/Football	Sport	Right	9	I

16	M	23	Footballer	Sport	Right	10	IV

17	M	28	Basketball	Sport	Right	17	I

18	M	27	Manual Laborer	Fall	Right	9	III

Mean		23.5				14.9	

* Classification according to Schernberg 1984 [22]

### Surgical technique for Ilizarov frame application

**Figure 2:**
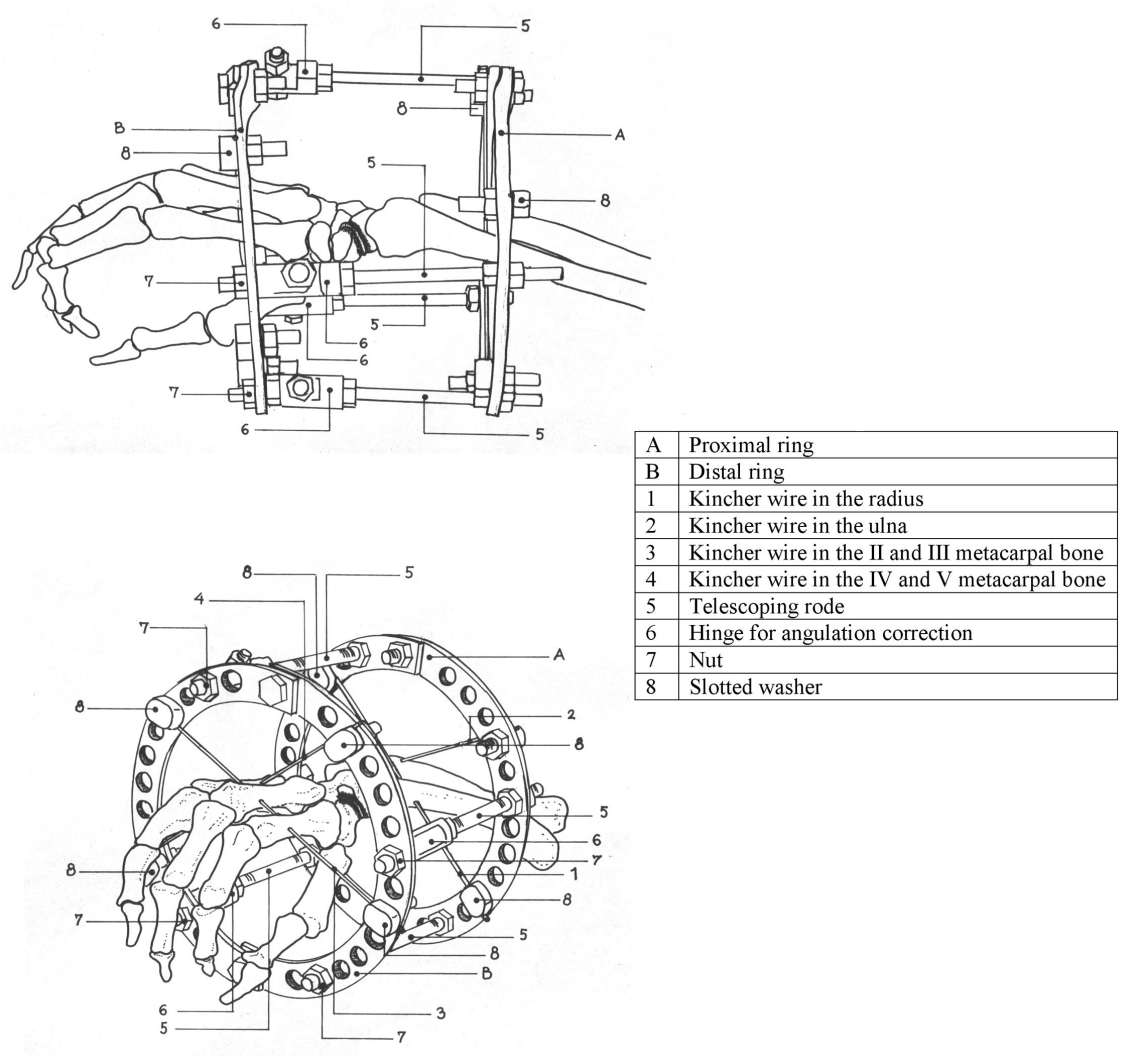
**An illustration of the Ilizarov device applied across the wrist: A-proximal ring, B distal ring, 1-Kirschner wire passed through the radius, 2-Kirschner wire passed through the ulna, 3-K wire in the 2^nd ^and 3^rd ^metacarpal bones, 4-K wire in the 4^th ^and 5^th ^metacarpal bone, 5-telescoping rode with 6-hinges joined together forming a complete hinge, 7-nuts and 8-slotted washers for K wire fixation**.

**Figure 3:**
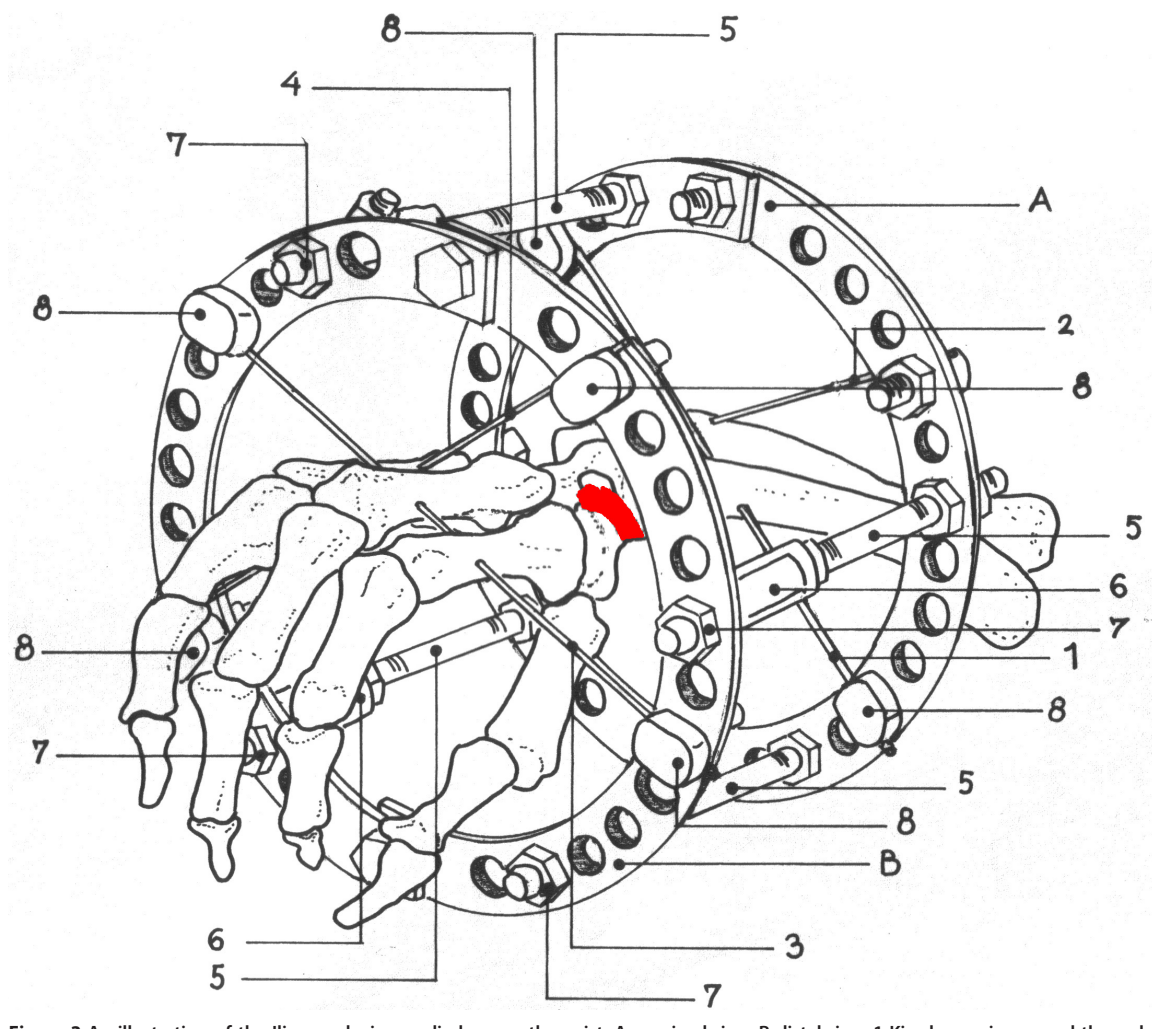
**An illustration of the Ilizarov device applied across the wrist: A-proximal ring, B distal ring, 1-Kirschner wire passed through the radius, 2-Kirschner wire passed through the ulna, 3-K wire in the 2^nd ^and 3^rd ^metacarpal bones, 4-K wire in the 4^th ^and 5^th ^metacarpal bone, 5-telescoping rode with 6-hinges joined together forming a complete hinge, 7-nuts and 8-slotted washers for K wire fixation**.

**Figure 4:**
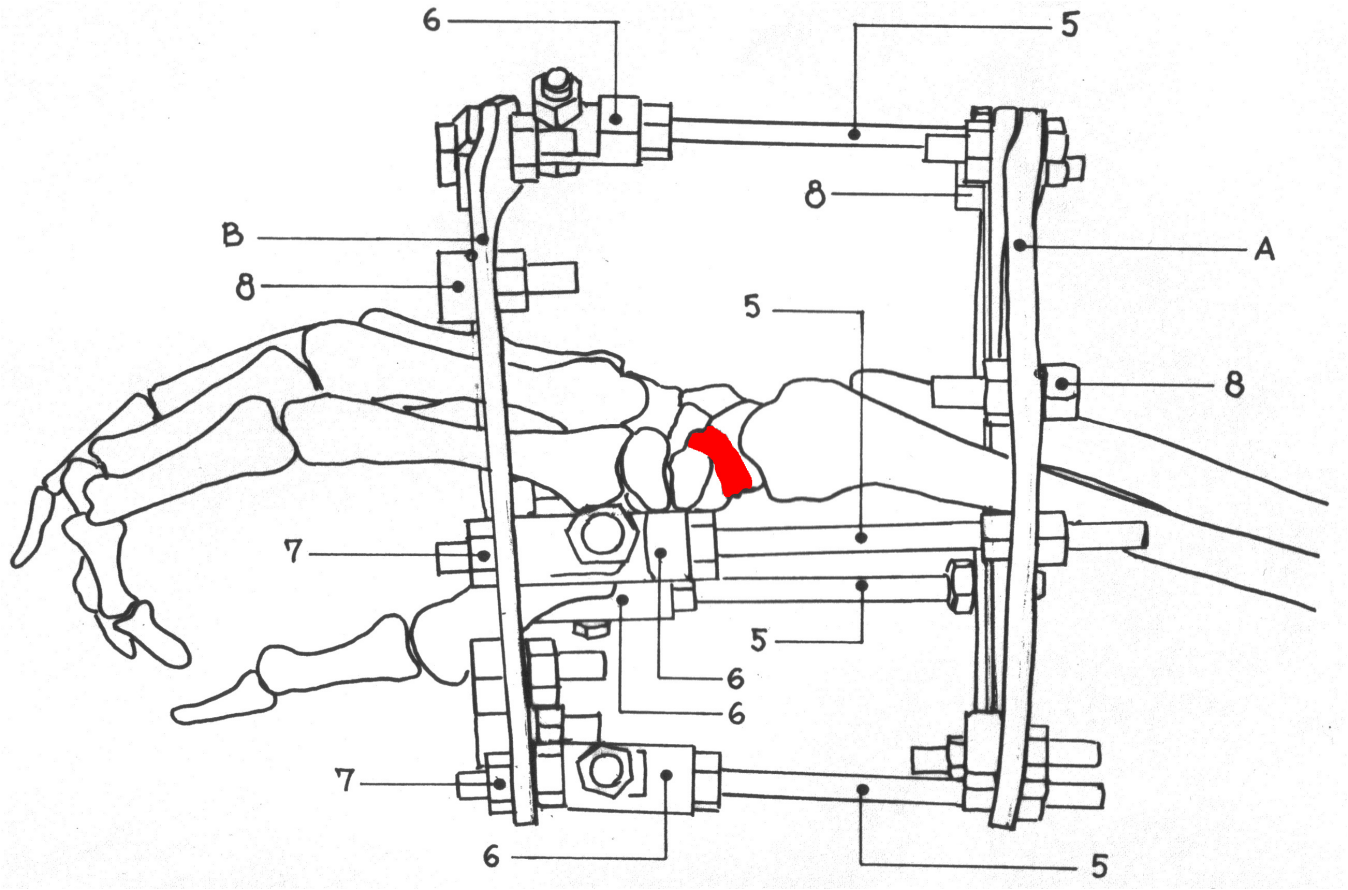
**An illustration of the Ilizarov device applied across the wrist: A-proximal ring, B distal ring, 1-Kirschner wire passed through the radius, 2-Kirschner wire passed through the ulna, 3-K wire in the 2^nd ^and 3^rd ^metacarpal bones, 4-K wire in the 4^th ^and 5^th ^metacarpal bone, 5-telescoping rode with 6-hinges joined together forming a complete hinge, 7-nuts and 8-slotted washers for K wire fixation**.

Patients were operated without tourniquet under regional anesthesia, with the arm placed volarly on a side table. The non-union site was not violated. The Ilizarov frame (Figures 2, 3 and 4) consisted of two rings (A and B) connected to one another with four threaded rods (diameter 3.5 mm, length 120 mm) and to the hand with non-threaded K-wires (diameter 1.55 mm). A circular frame was utilized in preference to a unilateral low-profile fixation device in order to be able to apply symmetrical distractive and compressive forces across the SNU site, in accordance with standard Ilizarov philosophy. The two proximal K-wires (#1 and #2) passed through the radius and ulna 3-5 cm proximal to the radiocarpal joint line. The K-wire passing through the radius (#1) was oriented from the volar to the dorsal side at an angle of 30 degrees in the frontal (coronal) plane, to avoid the radial artery. The K-wire passing through the ulna (#2) was oriented from the dorsal to the volar side at an angle of 30-45 degrees in the frontal plane, and exited the skin 2-3 mm from the tendon of the flexor carpi ulnaris muscle. These two K-wires (#1 and #2) were attached to the proximal ring (A) (with slotted bolts #8 and nuts #7 on the opposite side of the ring) and tensioned to 90-100 kg. The two distal K-wires (#3 and #4) were placed through the middle third of the metacarpal bones; the first K-wire (#3) through the second and third metacarpals from the radial side, and the second distal K-wire (#4) through the fifth and fourth metacarpals from the ulnar side of the hand. These two distal K-wires (#3 and #4) were both placed at angles of 30-40 degrees to the coronal plane, and fixed to the distal ring (B) (also with slotted bolts and nuts on the opposite side of the ring) with 90-100 kg of tension. The rings were connected with four threaded rods (#5) through a hinge (masculine and feminine ends connected) system (#6).

### Three stage distraction-compression procedure

Distraction of the SNU was commenced on the second postoperative day with the wrist in a neutral position. The distal ring was distracted (nut #7) at a rate on 1 mm per day, for a mean of 10 days (range 7-14 days), until mini C-arm fluoroscopy showed a 2-3 mm opening at the nonunion site. Following this, the non-union site was compressed for 5 days, at a rate of 1 mm per day, with the wrist in 15 degrees of flexion and 15 degrees of radial deviation; in an attempt to compress along the scaphoid axis [27]. The third stage involved immobilization with the Ilizarov fixator for 6 weeks, after which the frame was removed without anesthesia and unrestricted daily intensive physical therapy implemented for around 1-2 months, as required. Thus patients wore their frames for periods of between 55 and 62 days in total, allowing the scaphoid to continue to consolidate following fixator removal.

**Figure 5:**
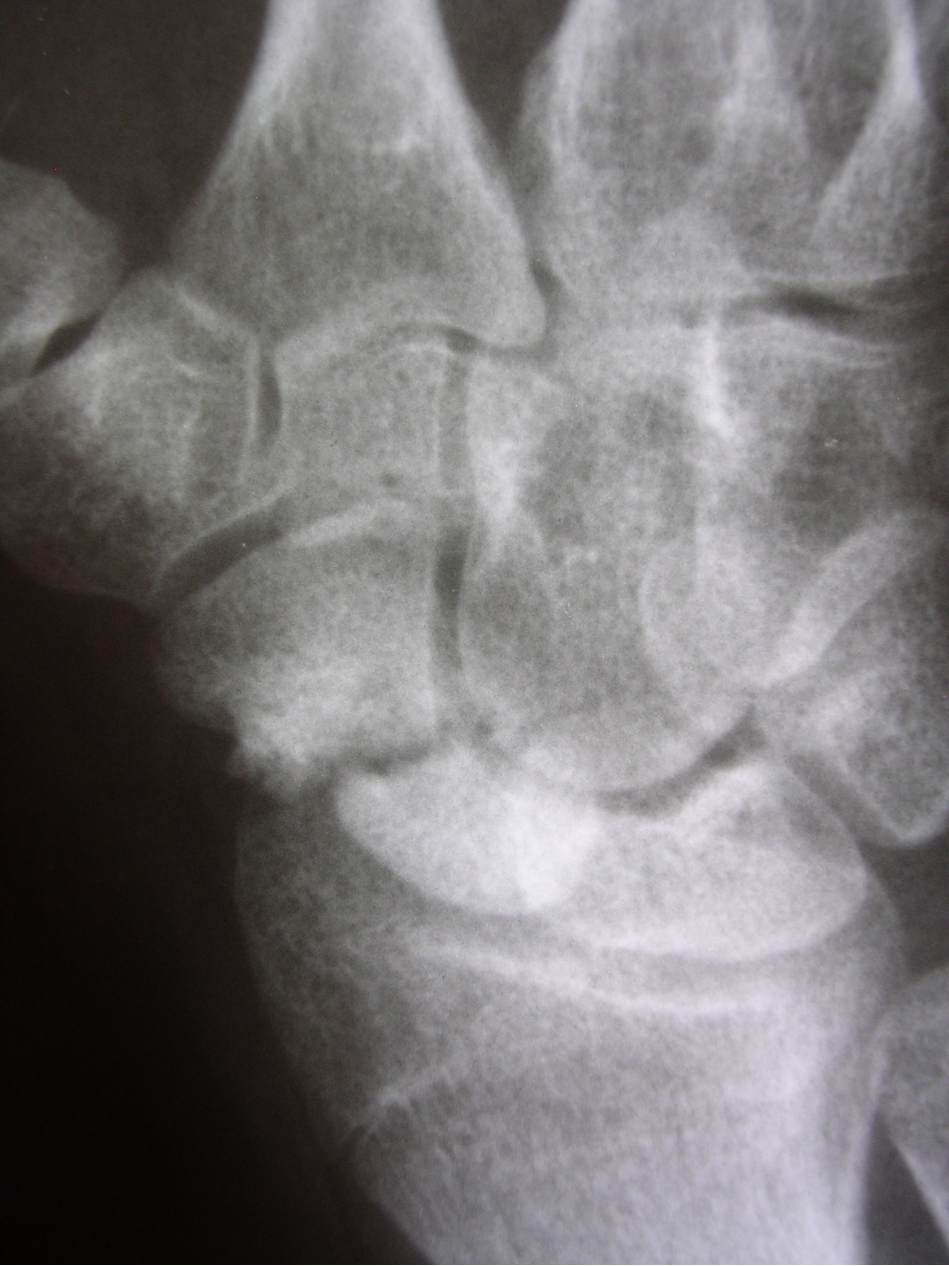
**Scaphoid non-union (SNU) in patient number 3, a preoperative radiograph**.

**Figure 6:**
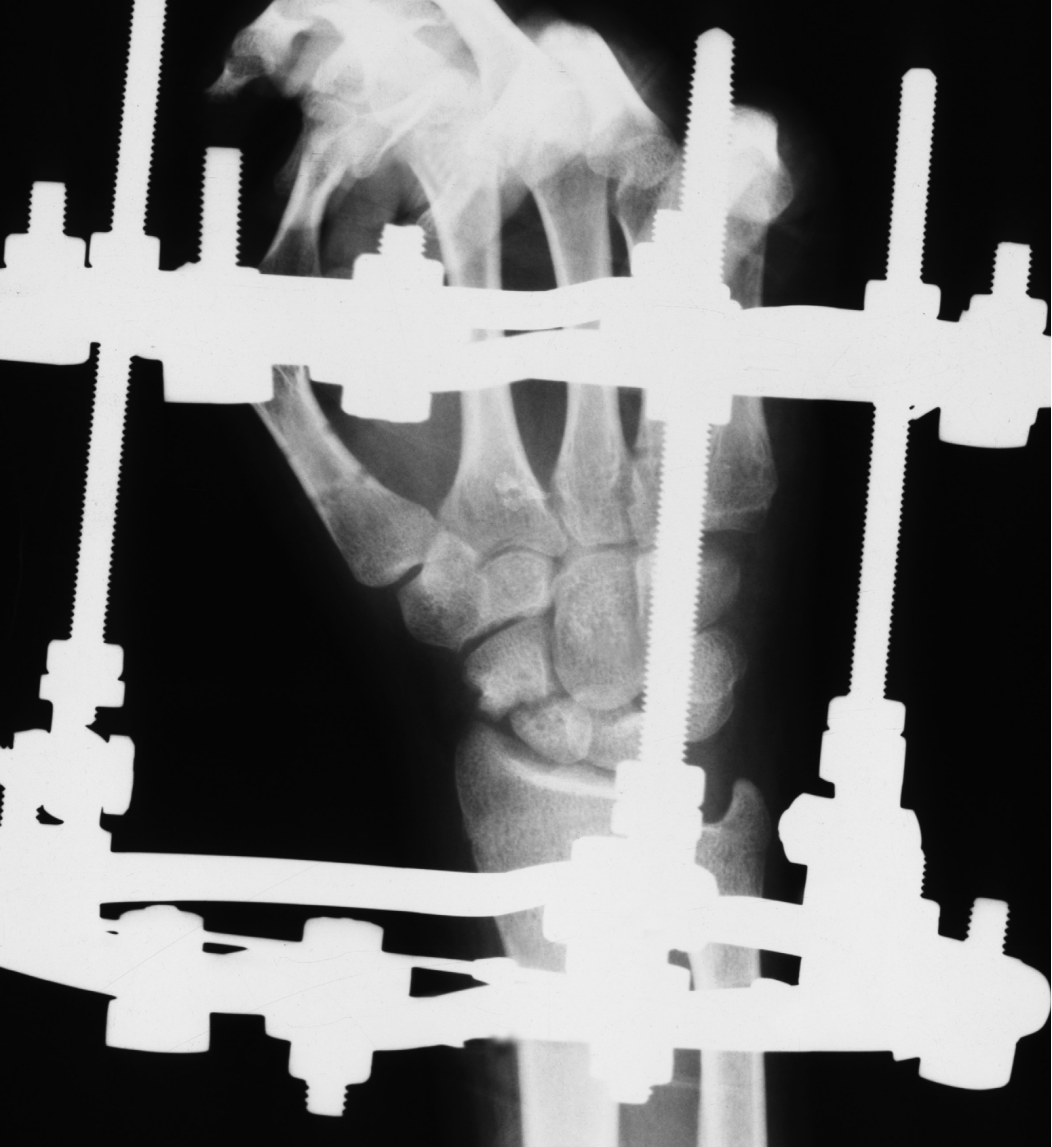
**A radiograph of the SNU in patient number 3 with the frame in situ**.

**Figure 7:**
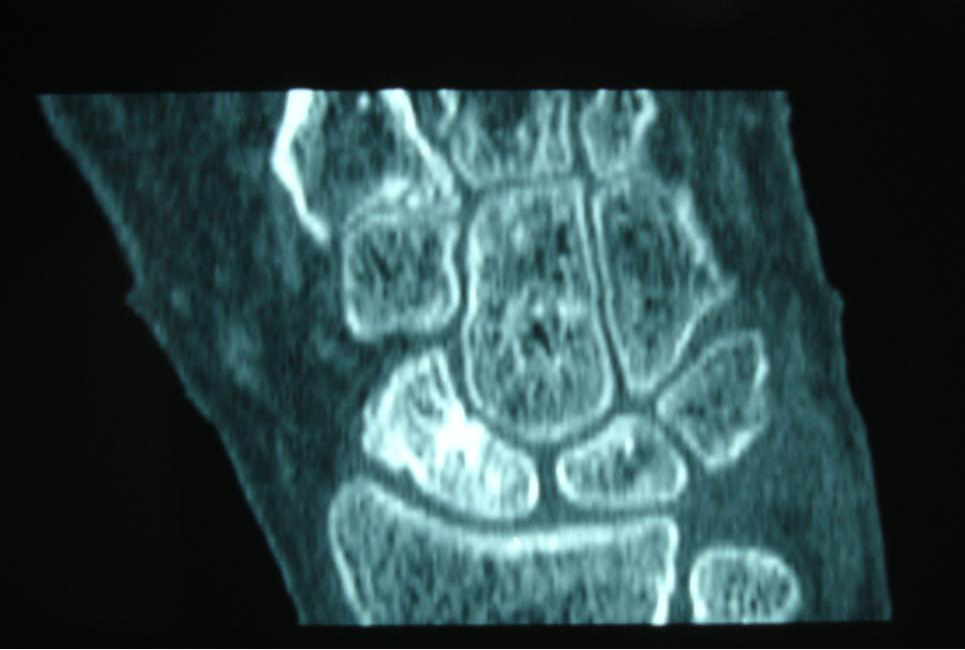
**A postoperative CT scan of the healed SNU in patient number 3**.

Patients were evaluated clinically and/or radiologically at 2-weekly periods following frame union, until bony union was achieved. They were also evaluated clinically at 6, 12 and 24 months post frame removal, with a mean follow-up of 37 months (range 24-72 months). Progression of healing was evaluated from conventional anteroposterior, lateral and scaphoid radiographs. Union was considered established when ossification and trabecular bridging was present between the distal and proximal fragments on x-ray. Thin slice CT scans were performed in each case to confirm the final radiographic union for the purposes of this study, and were evaluated by an independent observer (Figures 5, 6 and 7)[28,29]. Radiographs were also taken at 6 and 12 months following frame removal to identify any subsequent scaphoid collapse or other deformity.

The modified Mayo wrist score was used to evaluate the functional outcomes; this consists of the 4 categories: pain, the return to work or sporting activities, the range of wrist motion, and the grip strength, scoring a maximum of 25 points in each (total 100 points) (Table 2). The preoperative and postoperative modified Mayo wrist scores were compared by Wilcoxon's test of equivalent pairs. Grip strength of both affected and unaffected hands was measured using the Jamar dynamometer (Sammons Preston, Bolingbrook, Illinois).

**Table 2:** The modified Mayo wrist score (excellent 91-100, good 80-90, fair 65-79)

Item	Points	Definition
Pain	25	No pain

	20	Mild, occasional

	15	Moderate (tolerable)

	0	Severe, intolerable

Return to sport (work) at 6 months	25	Return without protection

	20	Return with protection

	15	Restricted return to sport, only exercises

	0	Unable to return to sport

Range of motion	25	90-100% (normal)

	20	80-89%

	15	70-70%

	0	50-69%

Grip strength	25	90-100% (normal)

	15	80-89%

	10	70-70%

	0	50-69%

## Results

Radiographic and clinical bony union was achieved in all 18 SNU patients after a mean of 89 days (range 70 - 130 days) (Table 3). There were no intraoperative complications and no injuries to nerves or vessels. Superficial pin-tract infections occurred in three patients and all resolved with local saline washes, occlusive dressings and oral antibiotic therapy. No patient developed complex regional pain syndrome (CRPS), digital tightness, stiffness, tendon adherence or contractures in either the MCP or IP joints. There was no loss of scaphoid height or collapse of regenerate bone noted radiographically following frame removal, and no patients developed a DISI deformity.

**Table 3:** The results of treatment for scaphoid non-union using the Ilizarov technique.

Case	Follow up time (months)	Return to work (days)	Wrist flexion (deg)	Wrist extension (deg)	Grip strength lbs(kg) injured side/contralateral side	Bone union/days	Pre-op Mayo score	Post-op Mayo score	Outcome Grade
1	82	110	80	70	80/90	90	15	100	Excellent

2	71	120	80	70	120/120	80	35	100	Excellent

3	64	100	80	70	110/115	90	0	90	Excellent

4	54	150	60	50	100/120	95	15	80	Good

5	53	90	80	70	80/80	70	50	100	Excellent

6	47	120	80	70	130/120	80	55	100	Excellent

7	41	95	70	60	110/120	75	15	85	Good

8	40	124	60	50	100/110	94	15	80	Good

9	37	100	50	40	80/120	100	0	60	Fair

10	37	105	70	60	120/120	70	45	90	Excellent

11	35	160	50	40	80/120	130	0	60	Fair

12	34	123	80	70	100/120	93	25	90	Excellent

13	31	117	70	60	110/110	87	25	90	Excellent

14	29	140	50	40	80/100	98	0	65	Fair

15	29	100	80	70	120/120	80	25	100	Excellent

16	27	115	60	70	120/120	90	30	95	Excellent

17	26	120	65	65	120/120	105	15	90	Excellent

18	24	100	70	70	100/110	94	25	85	Good

Mean	42.3	116.1	68.6 *	60.8	103(47)/113(51)	90.1	21.7	86.7	

*Mean flexion-extension arc was 129.4 degrees.

Taking measurements at the most recent follow-up, the mean postoperative modified Mayo wrist score was 86; significantly improved from the mean preoperative score of 21.3 (p < 0.01) (Table 3). Total flexion-extension wrist arc was 128.7 degrees, compared with 150 degrees in the uninjured hand; in only three patients (12, 14, 17) was there a restriction of movement more than 20% from the range of motion of the contralateral wrist. Mean grip strength was 101 lbs (46 kg) compared to 116 lbs (53 kg) in the uninjuried hand (87%). Eight patients regained 100% strength when compared with the non-dominant contralateral side, seven were weaker by 15-20%, and 3 patients were 20-30% weaker (Table 2 and 3). The results were classed as excellent in ten cases, good in four and fair in four according the modified Mayo scoring system. Fourteen patients were completely pain-free, and four patients had only occasional mild pain. All patients were able to return to their pre-injury occupations and levels of activity, following intensive physiotherapy, at a mean of 117 days (range 90-160 days) following the index operation. A mean of 5 sets of radiographs, 9 daily mini C-arm fluoroscopies and 1 CT scan were performed on each patient during the entirety of their treatment [30].

## Discussion and conclusion

There is currently no panacea for the successful treatment of SNU, with failures occurring in up to 25% of cases [3,10]. The main predictor for failure has been identified as the time elapsed between the initial injury and the treatment of the established SNU, with the success rates decreasing to 62% after delays of 5 years [3]. To achieve clinical and radiological union the following principles have been previously proposed: (i) preservation of the blood supply; (ii) bone grafting to achieve the original bony alignment and correct any humpback deformity; (iii) stable internal fixation and correction of carpal instability; and (iv) the treatment of SNU before the development of degenerative change [6,7,9].

To this end, past SNU treatments have included bone grafting with or without internal fixation. Stable internal fixation with AO or Herbert screws has been shown to improve union rates when compared with K-wire fixation [9]; a quantitative meta-analysis has reported overall union rates of 94% following screw fixation with bone grafting, compared with 74% following K-wire fixation [9,31]. The introduction of vascularized bone grafts has now also expanded the possibilities for SNU treatment to include proximal pole AVN and previous failed surgery [18-20], and has further improved union rates (to over 90%), though the harvesting and interposition of a viable vascularized bone graft requires great skill, and the placement of the fixation device is also technically demanding [13]. Impressive results were also seen in a series of 15 SNU patients (7 fibrous unions and 8 nonunions) treated using an arthroscopically assisted percutaneous internal fixation without bone grafting at a mean of 8.5 months post-injury. 100% union rates and good clinical outcomes were seen at 14 weeks post procedure [10] though this technically challenging procedure, we feel, has the potential to cause further soft tissue damage and disruption to the local biology, in less experienced hands.

A recent systematic review reported union rates of 80% using bone graft without fixation, 85% using bone graft with fixation, and 91% using vascularized bone grafts [13].

In contrast, the Ilizarov technique performed in this series involved the application of a circular external fixator without the use of bone graft, and thus its main advantage was to eliminate the need to expose the nonunion site, avoid causing further soft-tissue damage, as well as avoiding the morbidity and technical difficulties of potential bone graft harvesting. We found that the use of this system was not particularly technically demanding, and would be fairly straight forward for surgeons trained in fine-wire fixator application.

The main disadvantages to this technique related to the size of the bulky apparatus and the prolonged immobilization of the wrist joint. Postoperative wrist immobilization, however, is advocated with most other fixation and treatment methods [3,9], with periods of up to 80 weeks [13], and no patient in our series required the frame in situ for more than 9 weeks. Following intensive physiotherapy all patients achieved improved arcs of movement and no patient developed CRPS. Imprudent wire placement has the potential to cause a temporary tenodesis of the digital tendons during the distal-ring fixation, or damage to the ulnar nerve or radial artery when placing the proximal-ring K-wires, though no patient in our series had any problems with digital tightness, stiffness, tendon adherence or contractures in the MCP or IP joints.

Our initial results are encouraging, with bony union achieved in all fifteen patients after a mean of 89 days (70-130 days), comparing favourably to other standard techniques (42-112 days) [10,15,16,19,20]. Mean Mayo wrist scores (86 points) were also similar to those scores achieved in patients with vascularized bone grafts (82-92 points) [18]. The patients tolerated the apparatus well, and though rather bulky found that they had good use of the operated hand with the frame in situ. The procedure had a low complication rate with 4 pin-tract infections in 3 patients which resolved with local saline washes, occlusive dressings and oral antibiotic therapy.

We noted that one patient in this series, with an SNU of 15 months duration, developed a humpback deformity of approximately 70 degrees during their Ilizarov treatment. The reasons for this remain unclear, though we postulate that it may relate to the compression having not been applied along the anatomical axis of the scaphoid, thus producing palmar angulation [27]. This however was not seen in the other cases, and in fact the patient had a good clinical outcome with a Mayo score of 80, good grip strength and flexion-extension arc; and united their scaphoid nonunion in 95 days.

Our retrospective study has obvious limitations. We did not include SNU cases with humpback deformity, carpal instability, carpal collapse, AVN, or marked degenerative changes; these would have predisposed to an adverse outcome and therefore our results might not be directly comparable to those of other SNU series in the literature. In addition, we did not randomize the patients and compare the Ilizarov technique with other established methods for the treatment of SNU; thus it is difficult to draw any strong conclusions as to whether this technique is preferable.

However, the results of this study are promising and demonstrate that distraction-compression using the Ilizarov method without the use of bone graft is a safe technique, and that in selected cases may be an effective way of managing scaphoid nonunion. Further investigation should help to define a potential role for this technique in the management of scaphoid nonunion as well as to determine the mechanism by which distraction and compression applied through the Ilizarov fixator achieves successful bony union.

## Competing interests

The authors declare that they have no competing interests.

## Authors' contributions

MB and ST conceived the study; MB, ST, AL operated on the patients; ZK and HDA independently reviewed the radiology; VB, AL and HDA drafted the manuscript. All authors read and approved the final manuscript
